# A Note on Adherent Pericardium

**Published:** 1909-11-27

**Authors:** Lytton Maitland


### The General Practitioner's Column.

[Contributions to this Column are invited, and, if accepted, will be paid for.]

### A NOTE ON ADHERENT PERICARDIUM.

By LYTTON MAITLAND, M.B., B.S.

Marked hypertrophy of the heart is commonly to be assigned to one of the following causes: (1) valvular disease, (2) adherent pericardium, (3) arterio sclerosis, (4) chronic Bright's disease. A consideration of the clinical points of adherent pericardium allows the condition to be divided into three groups: (*a*) where it is of little importance to the patient, (*b*) where it is of serious import owing to cardiac symptoms, and (*c*) where it is associated with pleuritic and peritoneal symptoms.

It must be borne in mind that the importance of the pericardium is not due to its being a serous membrane but to the fact that it is a support to the heart, and prevents over-distension of that organ. When pericarditis occurs, this fibrous sac becomes softened, so that it no longer forms so strong a support to the heart; moreover the associated myocarditis further weakens the wall of the ventricle, and consequently dilatation occurs. Since the amount of work which the ventricle has to do increases in proportion to its cubic capacity, it follows that hypertrophy also will soon result.

Now when the adhesions consequent on an attack of pericarditis are forming, it is of the utmost importance to the patient that the heart should not be dilated. If tough adhesions form when the heart is dilated, they will hold it out and prevent it ever contracting again; it will remain dilated, and the patient will be permanently invalided. The prognosis is often made worse by the occurrence of adhesions on the outside of the pericardium to the pleura. On the other hand, if the heart is not dilated the presence of a universally adherent pericardium is a matter of comparatively little importance. This has been shown by post-mortem room findings. Cases have been met with in which the two layers of the pericardium were completely adherent, and in which there was no interference with the cardio-vascular mechanism.

It has also been observed that occasionally there is no hypertrophy, so that the presence of adhesions does not induce hypertrophy by resisting the contractions of the heart; nor is there anything in the statement, that the adhesions drag out the heart and thus induce hypertrophy. On the contrary, the adhesions may sometimes be torn or greatly stretched by the contraction of the heart.

In the second group of cases, the serious symptoms are caused because the adhesions were formed when the heart was dilated; in these cases there are not uncommonly adhesions also to the pleura. The symptoms are those of mitral regurgitation; the dilatation of the heart is accompanied by widening of the mitral orifice. This may lead to œdema of the lungs, cyanosis, nutmeg liver, anasarca, and renal trouble. In a case presenting the features of mitral incompetence, which does not seem to be benefited by rest in bed and digitalis, it may justifiably be inferred that the patient has possibly an adherent pericardium. A moderate dilatation usually is compensated by hypertrophy, but the amount of work to be done by the heart increases so rapidly in proportion to the dilatation, that often the hypertrophy cannot adequately compensate. Therefore it is obvious that in cases of acute pericarditis, attention must be concentrated on the prevention of dilatation. Anything which tends to raise the blood-pressure must be avoided on account of the softened pericardial support, and the concomitant myocarditis. Hence absolute rest in bed and no sitting up for any purpose is essential. The bowels must be kept relaxed to avoid straining, and the meals should be small and at frequent intervals to prevent flatulent distension of the stomach. Sleep must be secured by morphia if necessary; it is of the greatest value in this disease. Digitalis in some form or other, by prolonging the diastole (during which blood flows through the coronary arteries), will help repair of the heart muscle and will also tend to contract up the heart. Careful watch must be kept for any sign of nausea produced by the medicine, for that would tend to raise the blood-pressure; hypodermic administration of digitalis may be resorted to. Lastly, treatment must be prolonged.

This affection is, unfortunately, commoner in children than adults, and the prognosis is also more serious. The adhesions prevent due growth of the heart, while the pericarditis is more acute, accompanied by a greater degree of myocarditis, and the softened pericardium yields to a greater extent.

The third group of cases comprises those much rarer ones in which the peritoneal symptoms are prominent. There are present ascites and œdema of the legs with great dyspnœa. Post-mortem is found a condition known as polyserositis, in which the peritoneal covering of the liver is greatly thickened and there are numerous adhesions in the abdomen and in the pleural cavity, as well as universal pericardial adhesions.

